# Through thick and thin: The vaginal microbiome as both occupant and healer

**DOI:** 10.1371/journal.ppat.1013346

**Published:** 2025-07-22

**Authors:** Mart Sillen, Sarah Lebeer, Patrick Van Dijck

**Affiliations:** 1 Laboratory of Molecular Cell Biology, Institute of Botany and Microbiology, KU Leuven, Kasteelpark Arenberg, Leuven, Belgium; 2 Department of Bioscience Engineering, Research Group Environmental Ecology and Applied Microbiology, University of Antwerp, Groenenborgerlaan, Antwerp, Belgium; 3 Leuven One-Health Institute, KU Leuven, Leuven, Belgium; University of Maryland, Baltimore, UNITED STATES OF AMERICA

The vaginal microbiome (VMB) is the site-specific microbial community inhabiting the cervical and vaginal mucosa. It comprises less than 10% of the total human microbiome, although small in total quantity, the VMB plays an outsized role in local health. It is essential for mucosal immunity, infection prevention, and reproductive health, contributing to protection against sexually transmitted infections (STIs), regulation of inflammation, and favorable pregnancy outcomes. These roles place it at the forefront of women’s health research and innovation.

## A diverse community: The composition of the vaginal microbiome

The VMB represents a small yet functionally critical component of women’s total microbial cell population, though estimates vary with sequencing methodology and reference databases [1]. Bacteria, predominantly lactobacilli, are the most abundant members, however, the niche also includes fungi, viruses, archaea, and protozoa, many of which remain undercharacterized. Despite high interindividual variability and temporal fluctuation, the VMB plays a pivotal role in maintaining mucosal defense, immune modulation, and protection against urogenital infections [2].

The concept of a “healthy” VMB is context-specific, shaped by factors such as biogeography, hormones, genetics, behavior, and environment [3]. Amplicon sequencing has enabled classification into five main Community State Types (CSTs), with CSTs I, II, III, and V dominated by different *Lactobacillus* species (*L. crispatus, L. gasseri, L. iners,* and *L. jensenii/L. mulieris,* respectively), and CST IV characterized by diverse anaerobes including *Gardnerella* and *Prevotella* spp. [4]. CSTs offer a clinical framework for stratifying individuals by dominant microbial profiles, aiding in identifying those who may benefit from probiotic or antibiotic therapy [5]. However, many exhibit transitional or mixed states that do not fit into one CST [3]. Even expanded classifications, up to seven CSTs and 13 subtypes, fail to capture the microbiome’s dynamic nature [6]. To address this, topic modeling has been introduced. Rather than assigning samples to a single dominant type, topic models identify co-occurring taxa patterns, enabling detection of longitudinal changes and finer substructures [7]. CSTs and topic models serve different purposes: CSTs categorize people or samples to assess risk or guide therapy, while topic models uncover microbial patterns across samples.

Although *Lactobacillus*-dominant communities are generally linked to positive outcomes, not all non-*Lactobacillus* profiles signify dysbiosis, as some asymptomatic women harbor anaerobes like *Gardnerella spp.*, *Fannyhessea vaginae*, *Bifidobacterium spp.*, and *Prevotella spp.* [3]. While definitions of a “healthy” VMB are context-dependent, *Lactobacillus*-rich CSTs are consistently associated with a lower risk of bacterial vaginosis, STIs, and preterm birth [8]. In contrast, CST IV and other anaerobe-dominated states correlate with higher clinical risk [9]. The underlying mechanisms, such as lactic acid production, immune modulation, or microbial metabolites, remain poorly understood.

While bacteria dominate the VMB, the fungal component, or vaginal mycobiome, also contributes to local homeostasis [10]. *Candida* species are the most frequently detected fungi, with *C. albicans* present in 10–20% of asymptomatic women [11]. Non-*albicans Candida* species, including *C. glabrata* (*Nakaseomyces glabratus*), *C. krusei* (*Pichia kudriavzevii*), *C. parapsilosis*, and *C. tropicalis*, are less common but can proliferate under certain conditions [11]. Other taxa, such as *Cladosporium* and *Davidiella*, have also been identified via culture-independent methods [12]. Although often commensal, *C. albicans* and related species can become opportunistic pathogens, triggering symptomatic infections like VVC under specific host or environmental shifts [13].

Studying the vaginal mycobiome is challenging. Culture-based methods favor easily culturable fungi such as *C. albicans*, underestimating diversity [11]. Culture-independent approaches (e.g., ITS sequencing) improve detection but are limited by low biomass, contamination, and incomplete reference databases [14]. These methods provide relative abundance data, obscuring total microbial load shifts [15]. qPCR provides absolute quantification, allowing more accurate tracking of microbial dynamics. Integrating both approaches improves resolution and clinical relevance in VMB research.

These findings underscore the complexity and individuality of the VMB. While lactobacilli dominate, fungi also contribute to homeostasis and disease risk. Defining a “healthy” vaginal ecosystem thus requires personalized, context-specific interpretation.

## A dynamic ecosystem: The vaginal microbiome in flux

**Fig 1:**
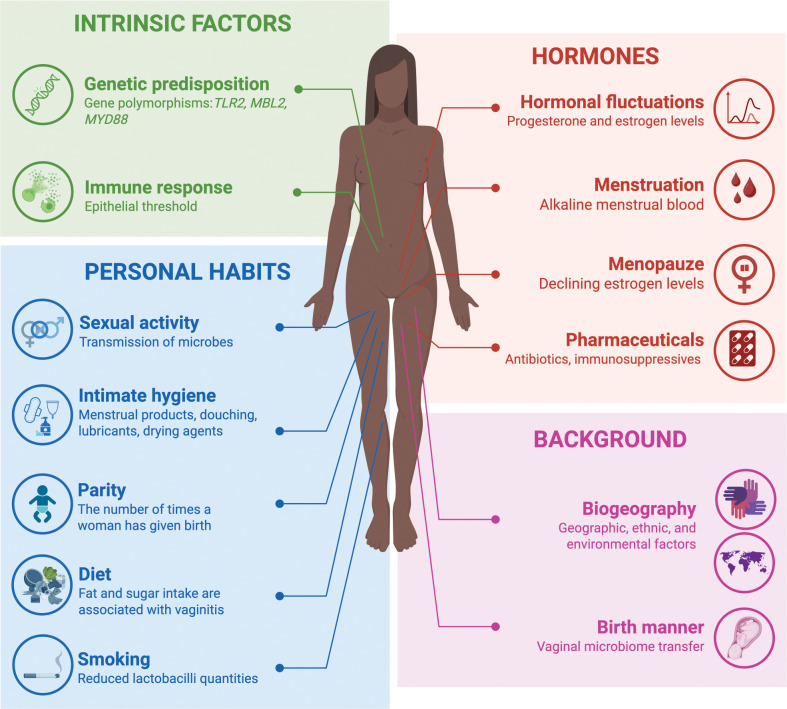
Multifactorial influences on the vaginal microbiome. This figure illustrates the intrinsic, hormonal, behavioral, and background factors that shape the structure and function of the vaginal microbiome. Host genetics and immune responses, hormonal fluctuations, personal habits (e.g., hygiene, sexual activity, diet, smoking, parity), and biogeographical context all influence microbial composition and resilience. These factors act in concert, modulating susceptibility to dysbiosis and shaping individual trajectories of vaginal health. Figure created with BioRender.com [3,4,17–21].

Beyond its interindividual variability, the VMB is a dynamic ecosystem that responds to internal and external cues through a mutualistic host–microbe relationship (Fig 1): the host provides a nutrient-rich mucosal niche, while microbes support epithelial integrity and local immunity [16]. The Isala Project, a citizen science-driven project profiling over 3,000 women, linked age, parity, menstrual cycle phase, contraceptive use, sexual activity, and hygiene practices to shifts in microbiome structure and stability [3].

Host genetic influence interindividual differences in VMB composition and stability. A genome-wide association study in 171 Kenyan women linked polymorphisms in immune-related genes (e.g., *TLR2*, *MBL2*, *MYD88*, and *TIRAP*) to microbial diversity and dysbiosis susceptibility [22]. Further research is needed to clarify the functional implications of these associations.

Hormonal fluctuations, throughout puberty, the menstrual cycle, pregnancy, and menopause, play a central role in shaping the vaginal microbiota [3,23]. Estrogen promotes epithelial maturation and glycogen accumulation, which is broken down by host α-amylase and related bacterial enzymes such as pullulanases into simpler sugars, serving as substrates for *Lactobacillus* spp. (and other vaginal taxa present) [24]. Lactobacilli convert sugars into lactic acid, lowering vaginal pH and creating an acidic environment that inhibits pathogen overgrowth while supporting *Lactobacillus* dominance [24]. In estrogen-depleted states, such as menopause, reduced glycogen and lactic acid lead to higher pH, diminished *Lactobacillus* abundance, and increased microbial diversity, features often linked to dysbiosis [9,25]. These shifts are not inherently pathological but reflect the microbiome’s physiological responsiveness to hormonal cues.

Beyond intrinsic factors, external exposures and lifestyle behaviors significantly shape VMB composition. Sexual activity, particularly with new or multiple partners, may introduce exogenous microbes or alter the environment via semen [3,17]. Hygiene practices (e.g., menstrual products, douching, spermicides, lubricants, and vaginal drying agents), and hormonal contraceptives affect micorbial balance, with effects depending on formulation [3,23,26]. Additionally, diet, smoking, and psychosocial stress have been associated with reduced microbial resilience and increased prevalence of dysbiotic states, such as bacterial vaginosis [18,19]. While individual behavioral effects are modest (e.g., *R*^2^ = 0.003 for intercourse in the past 24 h), the Isala Project showed that lifestyle factors collectively explained 10.4% of VMB variation [3].

VMB composition differs globally in ways that are increasingly understood through the lens of biogeography, a framework encompassing genetic ancestry, cultural practices, environmental exposures, and socio-behavioral context [20]. *Lactobacillus*-dominated profiles are more frequently observed in women of Western European and East Asian descent, while more diverse, anaerobe-rich communities (e.g., *Gardnerella*, *Atopobium*, and *Prevotella*) prevail in sub-Saharan Africa, Latin American, and Southeast Asian women [4]. Cohort studies confirm these trends but may reflect methodological or demographic biases [3,27]. Recognizing this diversity is essential to avoid one-size-fits-all definitions of vaginal health.

Early-life exposures shape initial VMB colonization. Vaginal delivery facilitates the vertical transmission of maternal microbes, promoting early colonization by *Lactobacillus* and other beneficial taxa [21]. In contrast, cesarean section favors colonization by skin and environmental microbes. Although direct evidence is limited, early colonization may influence the long-term trajectory of the VMB.

## When balance breaks: Vaginal dysbiosis and disease

Although generally resilient, the VMB can be disrupted by sustained and acute perturbations, such as hormonal shifts, hygiene practices, medication, or sexual activity, allowing opportunistic microbes to shift from commensal to pathogenic states, a process known as dysbiosis. Dysbiosis underlies the most prevalent vaginal infections; bacterial vaginosis (BV, 40%–50% of cases), vulvovaginal candidiasis (VVC, 20%–25% of cases), and trichomoniasis (TV, 15%–20% of cases) [28]. These conditions often present with overlapping, nonspecific symptoms, such as discharge, itching, and irritation, complicating diagnosis and leading to frequent mis- or overdiagnosis when based solely on clinical presentation [28]. BV is typically marked by reduced *Lactobacillus* spp. and overgrowth of anaerobic organisms such as *Gardnerella* spp., *Fannyhessea vaginae* (formerly *Atopobium vaginae*), *Prevotella* spp., and *Sneathia* spp. [29]. VVC, most frequently caused by *C. albicans,* though non-*albicans* species are increasingly recognized, presents with thick, white discharge, vulvar irritation, and inflammation [30]. Trichomoniasis, caused by the protozoan *Trichomonas vaginalis*, is characterized by frothy, yellow-green discharge and may also present with discomfort or itching [31]. Importantly, these diagnostic labels do not always reflect distinct microbial signatures, and coinfections or transitional states are not uncommon, further highlighting the need for molecular diagnostics to complement clinical assessment.

Interestingly, the symptoms of these infections are not solely due to the pathogens themselves; the host immune response plays a significant role in symptom development [32,33]. In VVC, symptoms often arise not simply from *Candida* colonization, but from an exaggerated inflammatory response by the host [34,35]. Inflammation is typically triggered by *C. albicans* hyphal transition, which activates epithelial immune signaling and recruits neutrophils that fail to clear the fungus, resulting in tissue damage and exacerbated symptoms [33,36].

## Restoring balance: Probiotics as allies in vaginal health

Treating vaginal dysbiosis requires more than antimicrobial therapy, as symptoms often arise not solely from microbial burden but from an exaggerated or dysregulated host immune response. Effective treatment should adopt a multifaceted approach, targeting pathogen clearance, immune modulation, and VMB restoration. While systemic immunomodulators are not established, local corticosteroids have occasionally been used for severe inflammation, such as in aerobic vaginitis, though supporting evidence is limited [37].

Probiotics, live microorganisms that confer health benefits when administered in adequate amounts, are emerging as complementary tools to manage vaginal dysbiosis [38]. While antibiotics remain the primary treatment, they can disrupt beneficial microbes and promote resistance, particularly in recurrent or chronic infections [39]. Vaginally adapted *Lactobacillus* species (e.g., *L. crispatus*; *L. jensenii/L. mulieris*, *L. gasseri,* and *L. iners*) and related genera (*Lacticaseibacillus*, *Limosilactobacillus*, and *Lactiplantibacillus*) with broader ecological distributions, are promising probiotic candidates due to their ability to support mucosal homeostasis and inhibit pathogen colonization and their ability to survive and persist, at least temporarily in the vaginal niche [40–42]. Several clinical trials have explored their potential. Oral supplementation with *Lacticaseibacillus rhamnosus* GR-1 and *Limosilactobacillus reuteri* RC-14 altered vaginal microbiota in 64 women [40], though a follow-up study in 120 BV patients found no added benefit when combined with metronidazole, highlighting context- and patient-dependent effects. Intravaginal *L. crispatus* CTV-05 (Lactin-V) reduced BV recurrence in a phase 2 trial (*n* = 228) following antibiotic treatment and was shown to be safe in a prior phase 1 study [41,43]. A pilot trial in 20 women with acute VVC tested a vaginal gel with *L. rhamnosus* GG, *L. pentosus* KCA1, and *L. plantarum* WCFS1, relieving symptoms in 45% without rescue antifungals and reducing *Candida* levels comparable to fluconazole [42].

Yeast-based probiotics, particularly *Saccharomyces cerevisiae,* show promise for recurrent VVC treatment due to their compatibility with antibiotics. In a randomized controlled trial (*n* = 60), oral administration of *S. cerevisiae* CNCM I-3856 (daily for 4 weeks) resulted in the detection of the strain in vaginal samples in a subset of participants, suggesting potential gut-to-vagina translocation [44]. However, follow-up studies showed no consistent effect on VMB composition, likely due to baseline CST variability and menstrual-cycle-induced shifts, especially postmenstrual *L. crispatus* decline, which likely obscured any probiotic effects. These findings support the feasibility of translocation but highlight the need for cycle-aware, stratified trials. Given inter-individual variation in microbiome composition and immune responses, personalized strategies such as tailored probiotics to VMB transplants [45] may outperform one-size-fits-all solutions.

The protective effects of probiotics are multifactorial, strain-specific, and not yet fully characterized. In lactobacilli, mechanisms may include lactic acid production, bacteriocin secretion, epithelial adherence, competitive exclusion, and biofilm formation. By destabilizing pathogenic biofilms, which shield microbes from immunity and treatment, probiotics may enhance clearance [46]. *L. crispatus,* for example, reduces *C. albicans* biofilms by depleting essential amino acids [47], and some strains can integrate into biofilms, disrupting structure and increasing antimicrobial susceptibility [48]. This mechanism warrants further investigation. Although often linked to microbiome restoration, certain *Lactobacillus* strains have been shown to directly modulate cytokine expression *ex vivo*, suggesting immune effects independent of microbial shifts [49]. *Lacticaseibacillus casei* Shirota stimulates IL-12 via TLR2/NOD2, while *Lactiplantibacillus plantarum* CAU1055 suppresses pro-inflammatory mediators in macrophages [50,51]. Though promising, these findings are mostly preclinical and require validation in human vaginal health contexts.

*S. cerevisiae* counters *C. albicans* by competing for epithelial adhesion, inhibiting filamentation, and suppressing virulence factors like aspartyl proteases, thereby limiting epithelial damage and invasion [52]. Known for its gut immunomodulatory effects, *S. cerevisiae* also promotes anti-inflammatory signaling and mucosal immunity, suggesting similar therapeutic potential in VVC, where hyperinflammation drives symptoms [53].

Probiotic strategies offer a biologically informed approach to restoring vaginal health by supporting beneficial microbes, suppressing pathogens, and modulating immune responses. However, due to the complexity of the vaginal ecosystem, careful consideration of strain selection, host factors, and timing is crucial. Tailored, longitudinal clinical research will be key to unlocking their full therapeutic potential.

## From resident to remedy: Harnessing vaginal microbes as probiotics

Translating this complexity of probiotics into effective interventions requires understanding which microbial traits are most relevant in specific contexts. Strain selection now emphasizes ecological compatibility and functional benefits within the vaginal niche. A strain’s ability to thrive in its intended environment is essential for probiotic efficacy, as it determines successful colonization, metabolic activity, and overall therapeutic potential [54], highlighting the limitations of repurposing non-vaginal probiotics in this highly specialized environment [55].

Targeted selection strategies now focus on identifying strains with niche-specific functional traits [55,56]. The underlying premise is that native vaginal microbes are inherently better suited to local conditions, such as low pH, glycogen availability, and hormonal fluctuations, and are therefore more likely to persist and function optimally within this environment [57]. For instance, strains isolated from women with low infection incidence are considered strong candidates. One such example is *L. crispatus* CTV-05 (Lactin-V), derived from the vaginal microbiota of a healthy premenopausal woman. It was selected for its robust production of lactic acid and hydrogen peroxide, which help maintain an acidic pH and inhibit pathogens [41]. Similarly, *L. rhamnosus* GR-1, isolated from the female urethra, has shown clinical efficacy in combination with *L. reuteri* RC-14 [58], through mechanisms such as epithelial adhesion, immunomodulation, and suppression of *G. vaginalis* and *C. albicans*. However, not all vaginal isolates are equally effective; even strains originating from the same niche can differ substantially in functional traits [59]. Building on this foundation, genomic and transcriptomic technologies now enable refined probiotic selection by identifying genetic markers linked to critical traits such as mucin adhesion, acid tolerance, and antimicrobial production [60].

The VMB is not merely a passive inhabitant but a dynamic contributor to health, reflecting physiological states and shaping host responses. From shaping immunity to resisting infection and guiding therapy, it remains a key frontier in reproductive health. Through thick and thin, the microbiome persists, sometimes disrupted, but never irrelevant, and offers new hope for precision interventions that harness its power to heal from within.
